# On a Rare Form of Poisoning by Quinine

**Published:** 1871

**Authors:** A. Brayton Ball


### *On a Rare Form of Poisoning by Quinine.*

By A. Brayton Ball, M.D.

The more common symptoms of quinism, such as headache, tinnitus aurium, vomiting, prostration, etc., are familiar to every physician, but the occurrence of an erythematous rash, accompanied by œdema, and extending over the whole body, followed by desquamation, is so rarely a toxic effect of quinine, that I have not found any mention of it by such systematic writers on materia medica as Headland, Wood, Stille, Beck, Biddle, Waring, Royle, and Trousseau and Pidoux.

Briquet, in his monograph on quinine,* quotes Chevalier as having observed that workers in quinine were liable to various cutaneous eruptions ; but this effect is ascribed by Briquet to local irritation by the drug. In his numerous experiments, the author noticed no special effect upon the skin, except a very constant diminution in its temperature. Rilliet and Barthez record a case of desquamation, and Bouchut a roseola following the use of medicinal doses of quinine. The case which is the subject of this article occurred about a year since, in the practice of a physician of this city, who has kindly given me the notes from which the account is compiled. I am convinced that this form of quinism occurs more frequently than might be supposed, from the fact of its having been overlooked by systematic writers on materia medica. My object in publishing this case is to elicit similar reports from others, and thus to secure the recognition of such accidents by our text books on this subject.

On April 15th, 1870, Mr. A., a merchant of this city, who had previously enjoyed good health, was taken ill with febrile symptoms which lasted five days, and were thought by his physician to be of a malarial nature. In accordance with this view, quinine was prescribed in the form of two-grain sugar-coated pills, of which the patient took three on April 22d, two days after perfect recovery from the previous attack. On retiring at night, Mr. A. noticed that his hands were beginning to swell, and soon experienced severe burning and itching, which deprived him of rest. Towards morning, the feet became similarly affected. When seen on the 23d instant, the hands and feet were swollen and erythematous, the eruption being most conspicuous on the palms and soles, extending to the wrists and ankles, marked by a sharply-defined border, and resembling the eruption of scarlatina. There was no febrile disturbance, and the patient expressed himself as feeling very well, with the exception of the annoyance from the burning and itching. During the following day, the rash extended over the whole body, and on the fourth day the cuticle of the hands and feet began to desquamate. Desquamation shortly ensued upon the trunk and limbs. The cause of these singular symptoms being entirely unsuspected, the quinine was ordered to be resumed on April 30th, and the same night, after having taken three pills (six grains), the patient experienced toxic effects of still greater severity, viz.: high fever ; pains in the limbs; tongue heavily coated, clearing in a few hours to a dark red color ; burning and pricking of the hands and feet, with a return of the swelling and rash, which eruption extended over the trunk and limbs, and was followed by a second desquamation. From this second attack he failed to convalesce quickly, and went into the country, remaining there about a fortnight, during which time,

* Traite Therapeutique du Quinquina et de ses Preparations, Paris, 1853.

although (so far as can be ascertained) he took no more quinine, he occasionally suffered from burning and itching in his hands and feet, and on one or two occasions there was a slight return of the rash. On Monday, May 23d, having returned from the country in good general health, he took two more pills (grains ten), on his own responsibility—one at noon, and the other shortly before dinner. While eating his dinner, he experienced a peculiar tingling sensation in his tongue, which, in a few minutes, became so swollen as to interfere with articulation and deglutition. This symptom was soon followed by a rapid pulse, heat of skin, mental excitement, and incessant muscular tremors. The hands swelled, assumed the classical boiled-lobster hue; in short, he was again ill for four or five days, and desquamation ensued for the third time. The quantities of quinine stated as taken are accurately given, aud a subsequent examination of the pills remaining in the box showed that they contained only the sulphate of quinine with a little cinchonine and the usual sugar coating. As if to leave no doubt as to the relation between the symptoms and the cause assigned, it has been ascertained that Mr. A. had a similar attack about six years ago, which was regarded by his physician as scarlet fever, and that the attack came on after he had taken a few doses of the tincture of bark, which had been prescribed for him as a tonic.

I have been able to find but three cases recorded in full, which resemble that of Mr. A. They all appeared in the British Medical Journal of 1869 and 1870, and are given below:

Dr. Edward Garraway (British Medical Journal, October 9th, 1869), says: "I was called last month to a lady aged forty, in previous good health, who had been suddenly seized with œdema of the face and limbs, accompanied by an unusual erythematous rash. She had considerable uneasiness in the præcordia, and was in a state of great alarm. Certainly, there was sufficient cause, for she was greatly disfigured, and her arms felt ready to burst. Her idea was that she had been poisoned by a white powder which she had procured at a druggist's in mistake for quinine, and of which about a grain had been taken in a glass of wine. I taxed her with having eaten fungi, shell fish, decomposing cheese, and other unwonted articles of food, but she pleaded guilty to none of these things. On bringing me the remains of the white powder, it proved to be pure sulphate of quinine. I repudiated the idea of this having done her any harm. After three or four days, the œdema and the rash subsided, but the skin of the face scaled off, and there was peeling of the hands and feet, as after scarlatina. My patient remaining somewhat enfeebled, I unreflectingly ordered a quinine mixture by way of tonic. Two hours after taking the first dose (two grains), she sent for me, exclaiming, 'Oh, you have poisoned me with quinine again.' To my infinite chagrin and mortification, all the former symptoms recurred. I doubt if I have omitted prescribing quinine any day for the last twenty years—in this locality it is largely needed—and this is the first instance in which I ever recognized any ill effect, beyond headache, resulting from its administration."

Dr. J. C. Thorowgood (British Medical Journal, December 11, 1869), says: "Mr. Hemming's account of the lady who, after taking a few grains of quinine, came out in a rash resembling scarlatina, recalls to my mind the case of a young woman among my out-patients at the Victoria Park Hospital, who, at her second visit, told me I had been giving her quinine. It was true I had prescribed quinine for her, and she told me she knew it to be so from her having come out in a rash all over her body after two or three doses had been taken. What remained of the rash when I saw her reminded me of the measles, and she told me that on four, if not five occasions, when quinine had been prescribed, this same rash had invariably appeared."

Dr. R. Lightfoot (British Medical Journal, January 8, 1870), says: "In my professional note book, under the date of January 20, 1868, I find the following brief history entered, which is corroborative of the experience of Messrs. Garraway and Hemming. I looked up various text books, and wrote to different medical friends at the time, but, like Mr. Hemming, could obtain no information on the point. Mrs. W., aged forty-six, of a nervous, irritable temperament, had for some time been taking five grains of ammonio-citrate of iron thrice daily. On the morning of the above date, by mistake, a mixture was sent her containing citrate of quinine and iron, instead of her usual medicine. Within half an hour after she had taken the first dose, containing but half a grain of citrate of quinine and two grains of the combined salt, I was sent for, and on arriving, found the patient covered from head to foot with a bright, papular rash, accompanied with intolerable itching. In a few minutes the rash became diffused and erythematous. The itching preceded the eruption, and it was so violent that my patient had torn her night-dress in numerous places, so as to get more freely at her skin, which she had scratched so vigorously as to make it bleed profusely. She was in a state of great anxiety, and complained of sinking about the heart, headache, burring in the ears, and great thirst. Her face was very puffy, more especially about the eyelids and upper lip. Her pulse was 105, and wiry. She said at once that she knew she had taken quinine, mentioning having suffered in a similar way when it was given on a former occasion. As she was very excited, I gave her a draught containing twenty-five minims of tincture of henbane, also directing her to take an occasional sip of soda water, and to sponge the whole body with a weak alkaline lotion. The eruption soon began to disappear, and in three hours was gone. It was followed by slight desquamation on the succeeding two or three days. The other symptoms lasted with gradually diminishing intensity until the ensuing morning, patient then feeling free from all annoyance. The symptoms in this instance both came on more quickly, and after a much smaller dose of quinine, than they did in either of the two cases which have recently appeared in the Journal. It is to be noticed, too, that they disappeared more speedily."—*Medical Gazette.*

June 27th, 1870.

